# Hemophagocytosis in Experimental Visceral Leishmaniasis by *Leishmania donovani*

**DOI:** 10.1371/journal.pntd.0004505

**Published:** 2016-03-04

**Authors:** Ayako Morimoto, Satoko Omachi, Yasutaka Osada, James K. Chambers, Kazuyuki Uchida, Chizu Sanjoba, Yoshitsugu Matsumoto, Yasuyuki Goto

**Affiliations:** 1 Laboratory of Molecular Immunology, Department of Animal Resource Sciences, Graduate School of Agricultural and Life Sciences, The University of Tokyo, Bunkyo-ku, Tokyo, Japan; 2 Laboratory of Veterinary Pathology, Department of Veterinary Medical Sciences, Graduate School of Agricultural and Life Sciences, The University of Tokyo, Bunkyo-ku, Tokyo, Japan; The Ohio State University, UNITED STATES

## Abstract

Hemophagocytosis is a phenomenon in which macrophages phagocytose blood cells. There are reports on up-regulated hemophagocytosis in patients with infectious diseases including typhoid fever, tuberculosis, influenza and visceral leishmaniasis (VL). However, mechanisms of infection-associated hemophagocytosis remained elusive due to a lack of appropriate animal models. Here, we have established a mouse model of VL with hemophagocytosis. At 24 weeks after infection with 1 x 10^7^ *Leishmania donovani* promastigotes, BALB/cA mice exhibited splenomegaly with an average tissue weight per body weight of 2.96%. In the tissues, 28.6% of macrophages contained phagocytosed erythrocytes. All of the hemophagocytosing macrophages were parasitized by *L*. *donovani*, and higher levels of hemophagocytosis was observed in heavily infected cells. Furthermore, more than half of these hemophagocytes had two or more macrophage-derived nuclei, whereas only 15.0% of splenic macrophages were bi- or multi-nuclear. These results suggest that direct infection by *L*. *donovani* causes hyper-activation of host macrophages to engulf blood cells. To our knowledge, this is the first report on hemophagocytosis in experimental *Leishmania* infections and may be useful for further understanding of the pathogenesis.

## Author Summary

Anemia is one of the major clinical manifestations during visceral leishmaniasis (VL), whereas mechanisms behind this symptom remain elusive. To get a better understanding of the responsible mechanism(s), we have developed for the first time a mouse model of VL exhibiting anemia. Mice chronically infected with *L*. *donovani* had low hematocrit, hemoglobin and erythrocyte counts while having up-regulated erythropoiesis, suggesting hemolytic events due to infection. We propose here that hemophagocytosis is one of the hemolytic events associated with anemia in the infected mice. The spleen is the major place for hemophagocytosis; there, multinucleated giant cells heavily infected with amastigotes are markedly observed and are the major cell type phagocytosing erythrocytes. These results suggest that heavy infection of macrophages with *Leishmania* parasites triggers phagocytosis of erythrocytes resulting in anemia during murine VL. Because hemophagocytosis has been reported in human VL cases, reproduction of the pathology in mice may facilitate an understanding of the mechanisms leading to anemia during VL.

## Introduction

Visceral leishmaniasis (VL), also known as kala-azar, is caused by parasitic protozoa of the genus *Leishmania*. Countries endemic for VL include India, Bangladesh, Nepal, Brazil, Ethiopia and Sudan. It is estimated that there are 300,000 new cases of VL and 20,000 deaths annually (WHO, 2012). VL is characterized by clinical manifestations such as fever, weight loss, hepatosplenomegaly, and anemia. *Leishmania* parasites develop as promastigotes in the midgut of the infected sand fly. Once transmitted from sand fly to mammalian host through blood feeding, these parasites proliferate as amastigotes within macrophages in the spleen, liver, and bone marrow.

Hemophagocytosis is a phenomenon that macrophages or histiocytes engulf erythrocytes and/or leukocytes in the bone marrow, liver or spleen [1]. Under normal conditions, macrophages phagocytose only senescent or injured blood cells. In some cases, however, engulfment of new and intact blood cells by hyper-activated macrophages or histiocytes can be observed at high frequency [1]. This uncommon hemophagocytosis has been reported in auto-immune disease [2, 3], malignancy [4–6] and infections with virus including Epstein-Barr virus and influenza virus [7, 8], bacteria including *Salmonella* and *Mycobacterium* [9, 10], and protozoa including *Babesia* and *Leishmania* [11, 12]. Patients showing hemophagocytosis are often accompanied with other manifestations such as fever, pancytopenia, and splenomegaly, which are diagnostic criteria for hemophagocytic syndrome (HPS) or hemophagocytic lymphohistiocytosis (HLH) (1).

There are reports on hemophagocytosis in human VL patients [13–18]. In those reports, hemophagocytes engulfing red blood cells were observed in the bone marrow of VL patients [13–18]. Those hemophagocytes can be cleared after treatment with anti-leishmanial drugs, such as amphotericin B and sodium stibogluconate [13, 15–18]. VL patients representing hemophagocytosis are often diagnosed as HPS since the typical symptoms of VL, including fever, splenomegaly and lymphadenopathy, are also common in HPS [14–16]. This misdiagnosis sometimes delays the treatment of VL [14, 16].

In some infectious diseases hyper-activation of macrophages can be induced by the high levels of cytokines, such as interferon-γ (IFN-γ), produced in response to the causative pathogen [19, 20]. There is also a report that macrophages stimulated with IFN-γ and LPS become hemophagocytic [21]. However, the mechanisms that lead macrophages of VL patients to engulf their own blood cells have not been studied well. Examination of these cells in human VL patients is difficult because biopsy of bone marrow or spleen is a highly invasive procedure of some risk to the patients. Therefore, establishment of an animal model representing hemophagocytosis will facilitate an understanding of the underlying mechanisms of the phenomenon in VL cases. Indeed, animal models representing HPS have been established for some diseases and helped elucidate the mechanisms of hemophagocytosis [22–26]. However, reports on hemophagocytosis in experimental VL are lacking.

Here, we report the establishment of a mouse model of VL exhibiting hemophagocytosis in which we analyzed the infection status of the phagocytes. These results demonstrate the importance of direct infection of parasites leading to hyper-activation of the macrophages and resulting in acquisition of the hemophagocytic character. These results serve as the first step in elucidating the process of hemophagocytosis during VL.

## Materials and Methods

### Ethics statement

All animal experiments were reviewed and approved by the Animal Experiment Committee at the University of Tokyo (Approval No. P14-930). The experiments were performed in accordance with the Regulations for Animal Care and Use of the University of Tokyo, which were based on the Law for the Humane Treatment and Management of Animals, Standards Relating to the Care and Management of Laboratory Animals and Relief of Pain (the Ministry of the Environment), Fundamental Guidelines for Proper Conduct of Animal Experiment and Related Activities in Academic Research Institutions (the Ministry of Education, Culture, Sports, Science and Technology) and the Guidelines for Proper Conduct of Animal Experiments (the Science Council of Japan). Collection of peripheral blood was performed under anesthesia with isoflurane. At the end of the experiments, the animals were euthanized by exsanguination under anesthesia with isoflurane followed by cervical dislocation.

### Mice and parasites

Male BALB/cA mice were purchased from Japan Clea, Tokyo, Japan. All mice were maintained under specific pathogen-free conditions. The mice were used for experiments at the age of 6–8 weeks. *Leishmania donovani* promastigotes (MHOM/NP/03/D10; a gift from the National BioResource Project at Nagasaki University [27]) were cultured in medium TC199 (Nissui Pharmaceutical, Tokyo, Japan) supplemented with 10% heat-inactivated fetal bovine serum (Thermo Scientific, Waltham, USA) and 25 mM HEPES buffer (MD Biomedicals, France) at 25°C.

### Experimental infection, hematological analyses and autopsy

*L*. *donovani* promastigotes in late log or stationary phase were washed with phosphate-buffered saline (PBS: Nissui Pharm) by centrifugation at 1,600×*g* for 10 min and were resuspended with PBS at the concentration of 1 × 10^8^ cells/ml. Mice were infected with 1 × 10^7^ *L*. *donovani* promastigotes by intravenous injection into the tail vein. Blood was collected from the orbital sinus of mice under anesthesia with isoflurane (Pfizer Japan Inc., Tokyo, Japan) both 12 and 24 weeks after infection using heparinized capillary tubes (TERUMO, Tokyo, Japan). Hematocrit was determined by centrifuging the tubes at 15,000 × *g* for 10 min. Hemoglobin was measured following Zander‘s procedure [28], and the number of blood cells were counted by microscopic examination. For analysis of polychromatic erythrocytes, thin blood smears were prepared using the heparinized blood, followed by fixation with methanol (WAKO, Osaka, Japan) for 5 min and staining with 5% Giemsa solution (Merck KGaA, Parmstadt, Germany) for 25 min. The ratio of polychromatic erythrocytes to total erythrocytes was calculated through microscopic observation of the stained smears at 200× magnification.

After the blood collection for hematological analyses, cardiac puncture was performed on those same mice under isoflurane anesthesia to collect the whole blood. The mice were then sacrificed by cervical dislocation to collect the spleen, liver, and bone marrow. Serum was collected from the blood after centrifuging at 5,000 × *g* for 10 min and analyzed for erythropoietin level using a Mouse Erythropoietin Quantikine ELISA Kit (R&D Systems, Minneapolis, USA). Stamp smears of the spleen and liver were fixed for 5 min in methanol and stained for 25 min with 5% Giemsa solution. Amastigotes were counted by microscopic observation of the stained smear at 1,000× magnification, and Leishman-Donovan Units (LDU) were enumerated as the number of amastigotes per 1,000 host nuclei times the tissue weight in grams as performed in a previous study [29].

### Hematoxylin and eosin (HE) staining and Quantitative analyses of hemophagocytes

The tissues collected at the time of sacrifice were fixed with 20% buffered formalin (Sumitani Shoten Co., Ltd, Osaka, Japan) and embedded in paraffin. Four-micrometer thick sections were prepared from the paraffin-embedded tissues. The sections were dewaxed and stained with Mayer’s hematoxylin solution (WAKO) for 90 sec and rinsed in running tap water for 1 hour. Next, the sections were stained with eosin solution (MUTO PURE CHEMICALS CO., Ltd., Tokyo, Japan) for 2 min. In the HE-stained splenic sections, the number of infected macrophages, hemophagocytes, and multinucleated giant cells and the total number of splenic macrophages were counted in 5 random microscopic fields of the red pulp at 1,000× magnification. Also, around 100 hemophagocytes were individually analyzed in each section of the red pulp (1,000× magnification) for the number of host nuclei as well as the number of *L*. *donovani* amastigotes present. In this study, a macrophage was defined as a large cell (~20 μm in size, except for multinucleated giant cells) with large cytoplasm and round non-polymorphic nucleus. In contrast, hemophagocytes and multinucleated cells were defined as those cells containing engulfed red blood cells inside their phagosomal compartments and those cells with multiple round nuclei, respectively.

### Immunohistochemical analyses

Immunohistochemical staining was performed to characterize the subpopulation of heavily infected and multi-nucleated macrophages. Paraffin-embedded tissues were dewaxed and boiled in Tris-EDTA buffer (10 mM Tris Base, 1 mM EDTA solution, 0.05% Tween 20, pH 9.0) or 10 mM sodium citrate buffer (pH 6.0) for 20 min, followed by washing with tap water. Endogenous peroxidase was inactivated with 0.3% H_2_O_2_ in methanol for 30 min. After blocking with Block Ace (DS Pharm., Osaka, Japan), rabbit anti-mouse CD11b (Abcam, Cambridge, UK), rat anti-mouse F4/80 (AbD Serotec, Oxford, UK) or MOMA-2 antibody (rat monoclonal; Abcam) antibody was applied to the serial sections of spleens, and the sections were incubated for 1 h at room temperature and washed with PBS. Horseradish peroxidase (HRP)-conjugated anti-rat IgG (Nichirei Bioscience, Tokyo, Japan) or biotinylated anti-rabbit IgG (Nichirei) was applied, and the sections were incubated for 1 h at room temperature and washed with PBS. For the CD11b staining, alkaline phosphatase-conjugated streptavidin (Nichirei) was applied, and the sections were incubated for 1 h at room temperature. After enzymatic color development was performed using 3,3'-diaminobenzidine (Nichirei) or 4- [(4-amino-m-tolyl)(4-imino-3-methylcyclohexa-2,5-dien-1-ylidene)methyl]-o-toluidine monohydrochloride (new fuchsine, Nichirei), the sections were counterstained with Mayer’s hematoxylin solution for 1 min and rinsed with tap water.

### Statistical analyses

Statistical comparisons of means between naive and infected mice were performed by two-way ANOVA followed by Bonferroni’s multiple comparison test or unpaired *t* test with GraphPad Prism 6 software (GraphPad Software, Inc., La Jolla, USA). A difference between groups was considered as statistically significant when the *P* value was less than 0.05.

## Results

### Hemophagocytosis in the enlarged spleen of *L*. *donovani*-infected mice

**Fig 1:**
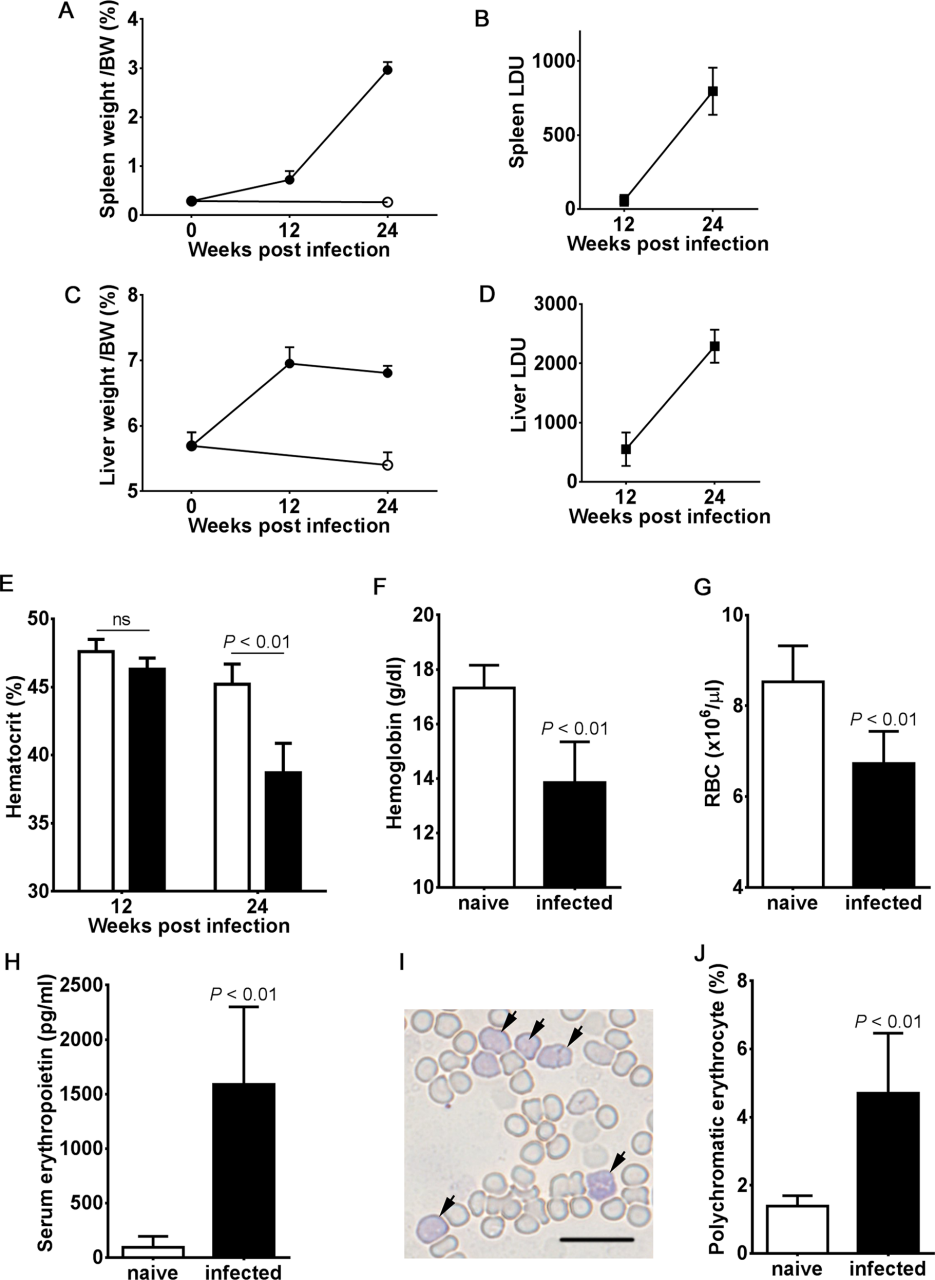
Anemia caused by *L*. *donovani* infection. Mice were infected with 1x10^7^ *L*. *donovani* promastigotes. After 12 and 24 weeks, infected mice as well as age-matched naïve mice were sacrificed to examine organ weights and parasite burden of the spleen (A and B) and liver (C and D), hematocrit (E), hemoglobin (F), peripheral blood red blood cell counts (G), serum erythropoietin levels (H), and proportion of polychromatic erythrocytes in peripheral blood (J). The open symbols or bars represent naïve mice and closed symbols or bars represent infected mice. The mean and SD of at least 3 mice in each group are shown. These are representative of three independent experiments with similar results. *P* values by two-way ANOVA followed by Bonferroni’s multiple comparison test (for E) or unpaired *t* test (for F, G, H and J) are shown. ns, not significant. Fig 1I shows representative microscopic images of Giemsa-stained smears of peripheral blood from infected mice. Arrows indicate polychromatic erythrocytes. Scale bar, 20 μm.

*L*. *donovani* infection induced hepatosplenomegaly in BALB/cA mice. The spleen and liver of the infected mice became significantly larger in size over time than those of uninfected mice. Mean weight per body weight ± SD of the spleen and liver from those infected mice were 0.72 ± 0.18% and 2.96 ± 0.16% at 12 weeks post-infection (p.i.), and 6.95 ± 0.25% and 6.81 ± 0.11% at 24 weeks p.i, respectively (Fig 1A and 1C). In contrast, those of the uninfected mice (age-matched to the 24 week-infected mice) were 0.27 ± 0.02% and 5.40 ± 0.20%, respectively. Parasite burdens in both tissues also showed increases from 12 to 24 weeks p.i. Means ± SD of LDU for the spleen were 58.9 ± 37.0 at 12 weeks p.i. and 796 ± 159 at 24 weeks p.i., and for the liver the means ± SD were 551 ± 282 at 12 weeks p.i. and 2,291 ± 279 at 24 weeks p.i. (Fig 1B and 1D).

Microscopic observation of HE-stained spleen at 24 weeks-infected mice revealed pathological changes compared with that of the uninfected tissues. The enlarged spleens from the infected mice were coupled with expansion of both red pulp and white pulp; nevertheless, both red pulp and white pulp were structurally intact. The red pulp showed more significant expansion than white pulp and was filled with an increased number of macrophages/histiocytes with a characteristic of diffuse histiocytosis. There were other myeloid cells including monocytes and neutrophils observed in the red pulp of the infected spleen, with macrophages/histiocytes constituting the major myeloid cells in the area and the predominant host cells for the observed amastigotes.

In addition to hepatosplenomegaly, the 24 week-infected mice exhibited anemia with lower hematocrit, hemoglobin and red blood cell counts (38.7 ± 2.16%, 13.9 ± 1.49 g/dl and 6.73 x 10^6^/μl, respectively) than the naive mice (45.2 ± 1.48%, 17.3 ± 0.83 g/dl and 8.53 x 10^6^/μl, respectively) (Fig 1E to 1G). Those infected mice had higher levels of serum erythropoietin compared with the naïve mice (1,589 ± 712 pg/ml vs. 94.3 ± 100 pg/ml) (Fig 1H). Furthermore, a higher frequency of polychromatic erythrocytes was observed in the peripheral blood of infected mice than of naïve mice (4.70 ± 1.76% vs. 1.39 ± 0.30%, respectively) (Fig 1J). At 12 weeks post-infection, no significant decrease in the above hematological parameters was found (Fig 1E).

**Fig 2:**
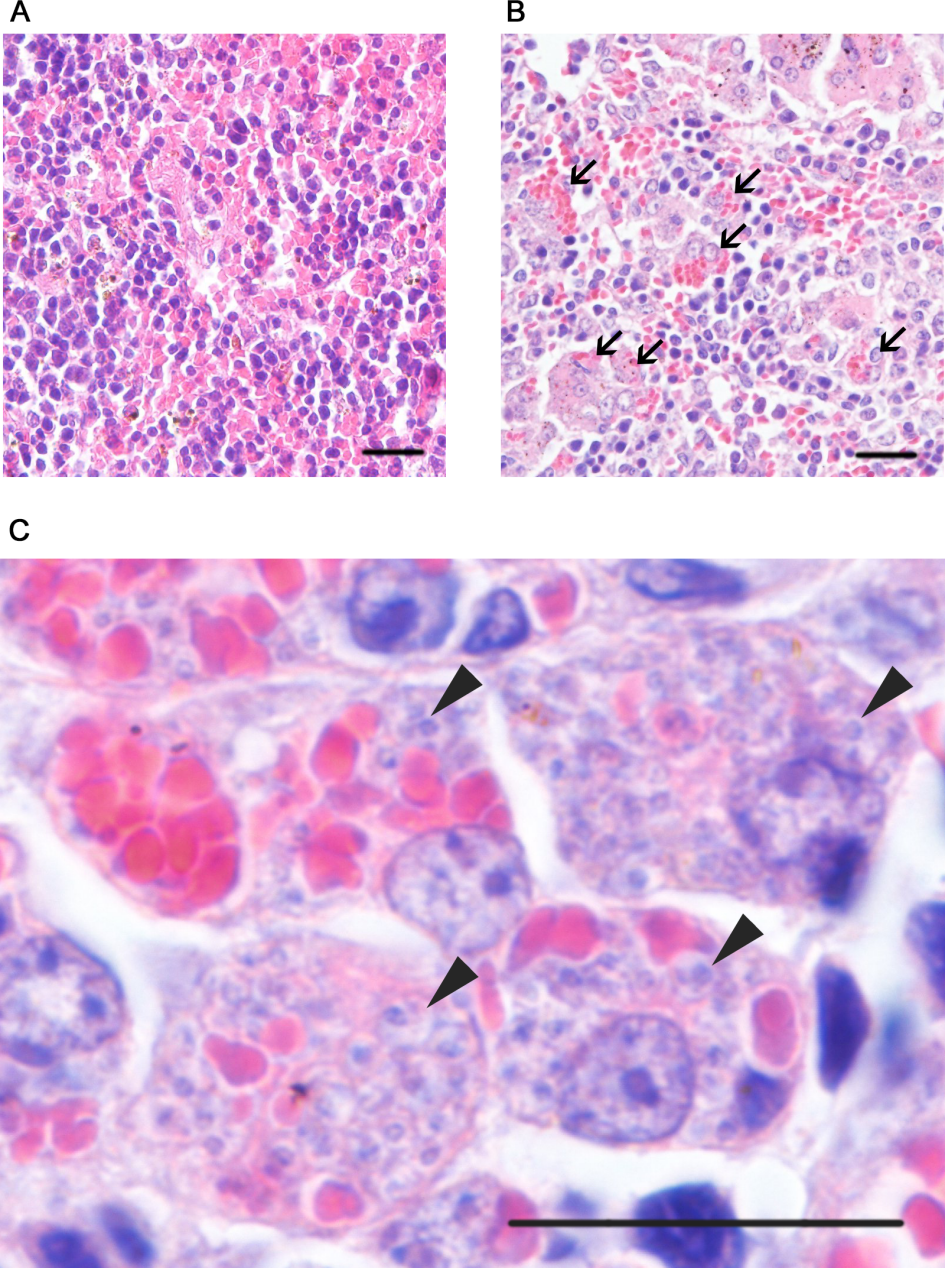
Hemophagocytosis in the spleen of *L*. *donovani*-infected mice. (A, B) Low magnification images of HE-stained sections of the red pulp of spleens from naïve (A) or *L*. *donovani*-infected mice (B). Hemophagocytes engulfing multiple erythrocytes (arrows) were found in the spleen of the infected mice, whereas such cells were not found in naïve mice. (C) A high magnification image of an HE-stained section of the spleen from infected mice. *L*. *donovani* amastigotes (arrowheads) were often found in hemophagocytes. Scale bars, 20 μm.

Next, the spleen of the 24 week-infected mice was examined for hemophagocytosis by microscopic observation of the HE-stained section. Erythrocytes were observed to be internalized in phagosomal compartments of the splenic macrophages in the red pulp (Fig 2). At 24 weeks, hemophagocytes accounted for 28.6% of the total splenic macrophages in the infected mice. In contrast, such macrophages were not observed in the spleen of uninfected mice. The liver and bone marrow of the 24 week-infected mice were also examined for the presence of hemophagocytes. Although amastigotes were detected in those tissues, there were less frequency of hemophagocytes in the bone marrow than the spleen and no detectable levels of hemophagocytes in the liver (S1 Fig). At 12 weeks of post-infection, hemophagocytes were less frequently observed; they were only 9.41% of the total splenic macrophage population.

### Heavy infection and giant cell phenotype as characteristics of hemophagocytes

**Fig 3:**
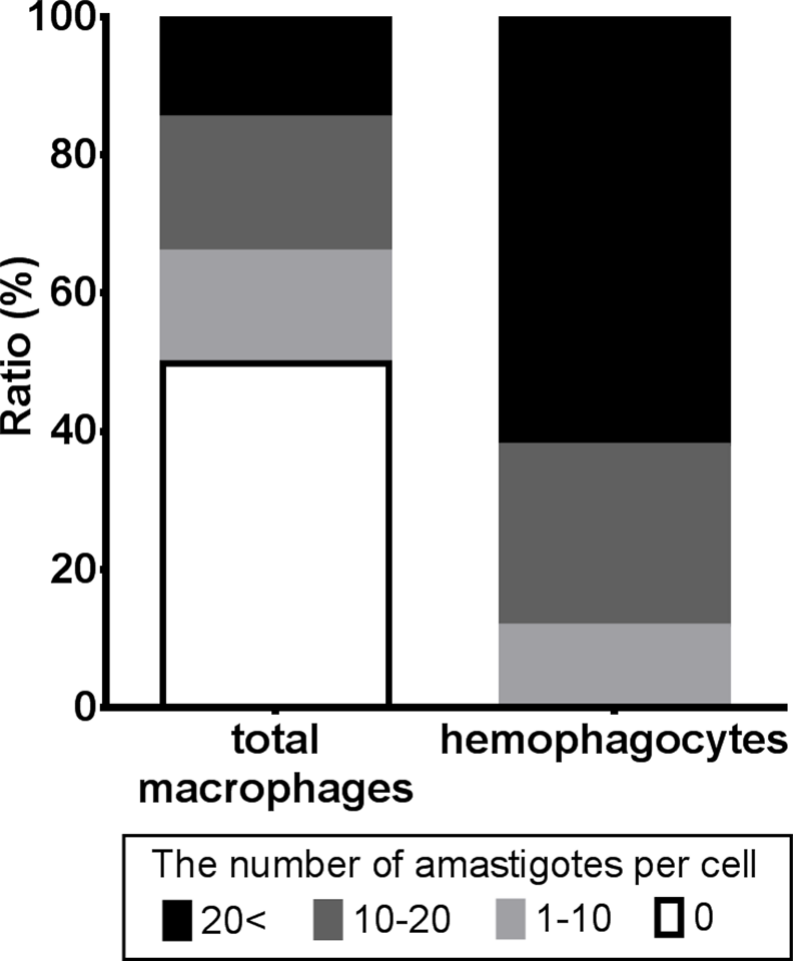
Heavy infection of hemophagocytes by *Leishmania* parasites. The number of amastigotes per macrophage/hemophagocyte in the spleen of *L*. *donovani*-infected mice was analyzed by microscopy of HE-stained tissue sections. Host cells were categorized, based on the number of parasites per cell, into the groups none (0 amastigotes), low (1–10 amastigotes), moderate (11–20 amastigotes) and high (more than 20 amastigotes). The graph shows percentages of cells with no, low, moderate and high infection in either total macrophages or hemophagocytes only.

Histological analyses on the spleens from the 24 week-infected mice also revealed that all of the hemophagocytes were infected with amastigotes although only about one-half (50.2%) of splenic macrophages were parasite infected (Fig 3). Furthermore, hemophagocytosis was observed more often in heavily infected macrophages. The infection status was also categorized based on the number of parasites per macrophage. The percentages of splenic macrophages with low (1–10 amastigotes), moderate (11–20 amastigotes), and high (more than 20 amastigotes) parasite infections, were found to be comparable (16.0 ± 1.7%, 19.4 ± 2.4% and 14.7 ± 1.5%, respectively) (Fig 3). In contrast, the majority of hemophagocytes were categorized in the high infection group (66.5 ± 6.2%), followed by moderate (26.2 ± 7.9%) and low (11.7 ± 3.7%) (Fig 3).

**Fig 4:**
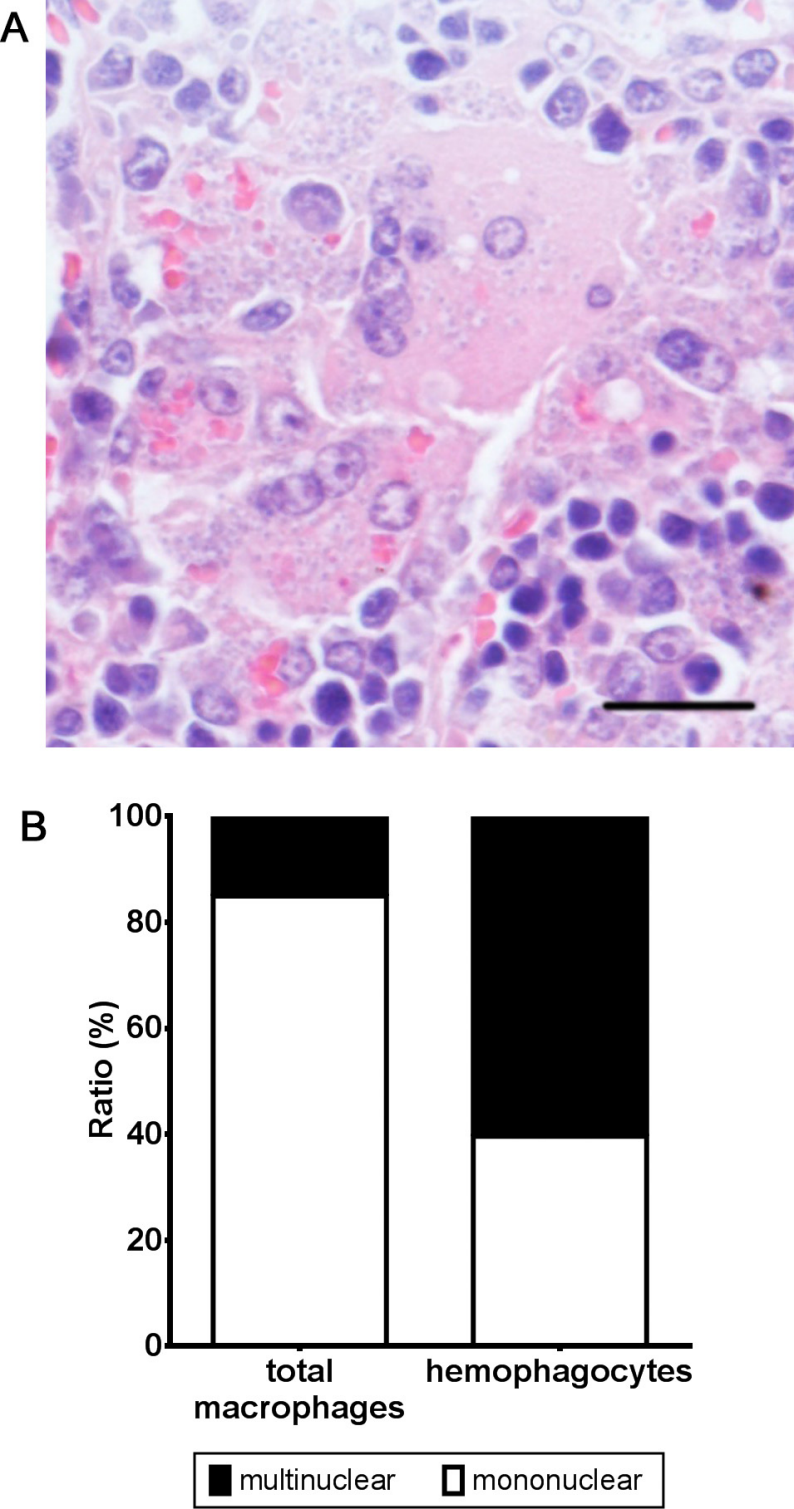
The multinuclear character of hemophagocytes in the spleen of *L*. *donovani*-infected mice. (A) A representative light microscopic image of MGCs engulfing erythrocytes in a thin section of a spleen from *L*. *donovani*-infected mice (24 weeks p.i.). Scale bar, 20 μm. (B) Proportion of mononuclear and multinuclear cells in the total macrophage and hemophagocyte only populations.

Besides having a heavy infection, the multinucleated giant cell (MGC) phenotype was prominent in those hemophagocytes (Fig 4A). The multinucleated macrophages accounted for 15.0 ± 6.2% of the total splenic macrophages (Fig 4B). No MGC were observed in spleens of uninfected mice. Although a few multinucleated macrophages were found in the liver or bone marrow of the infected mice, the ratio in those tissues was lower than that in spleen. The multinuclear phenotype was even more prominent in hemophagocytes where 60.4 ± 5.8% of splenic hemophagocytes of the infected mice were multinucleated (Fig 4B).

### Immunohistochemistry for characterization of heavily infected and multinucleated macrophages

**Fig 5:**
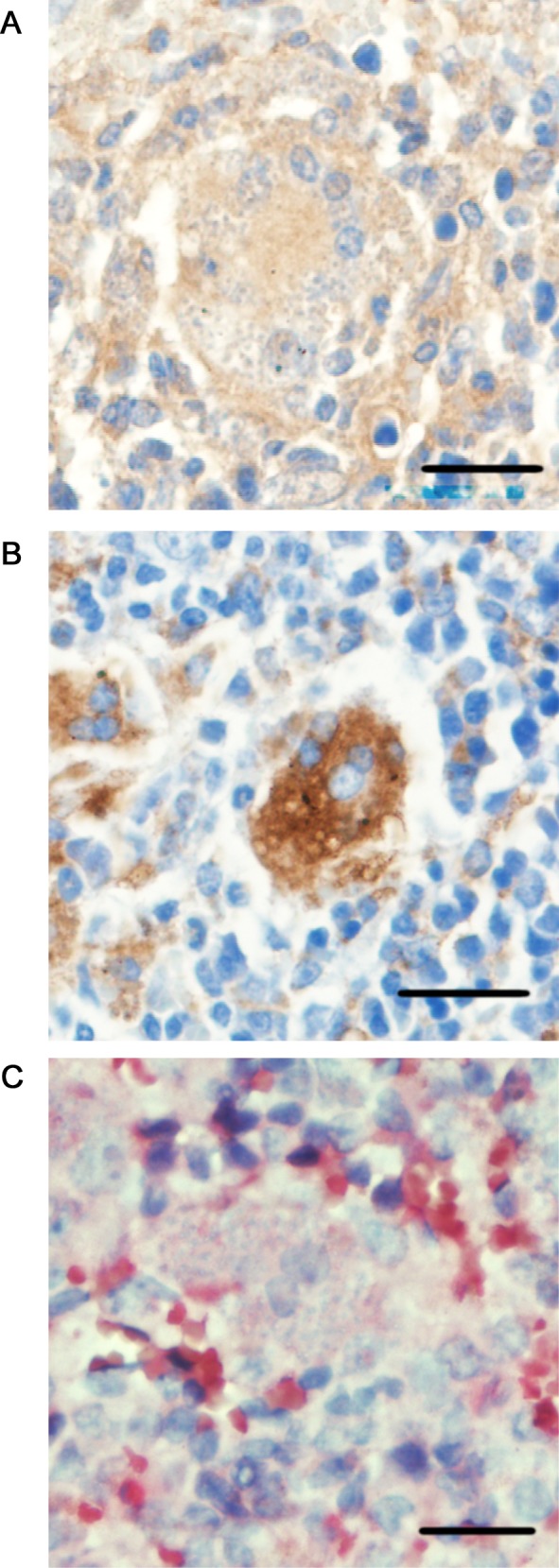
Characterization of heavily infected and multinucleated macrophages by immunohistochemistry. Sections of the spleen of *L*. *donovani*-infected mice at 24 weeks post-infection were stained with anti-F4/80 (A), MOMA-2 (B) or anti-CD11b (C) antibody, followed by counterstain with hematoxylin. Cells in the red pulp with positive signal are shown. Parasites are visualized as blue dots. Scale bars, 20 μm.

Immunohistochemical staining was performed on the spleen from uninfected or 24 week-infected mice by using anti-F4/80, anti-CD11b or MOMA-2 antibodies. F4/80 and CD11b signals were observed broadly in the red pulp of the uninfected spleen, whereas a MOMA-2 signal was detected only in a limited population of cells in both red pulp and white pulp (S2A, S2C and S2E Fig). MOMA-2-positive cells increased after 24 weeks of infection, and the increase was more prominent in the red pulp, whereas such an increase was not evident for F4/80 and CD11b (S2B, S2D and S2F Fig). The F4/80 staining of MGCs harboring amastigotes was positive and of similar intensity to the other surrounding cells, while MGCs were actually less intensely stained with anti-CD11b than other nearby positive cells (Fig 5A and 5C). Infected MGCs were MOMA-2-positive with a more concentrated staining pattern compared to surrounding non-MGCs (Fig 5B).

## Discussion

We have established for the first time an experimental animal model of *Leishmania* infection representing hemophagocytosis (Fig 2), a phenomenon observed in human VL cases. The reason for our success could be because few observers have monitored *Leishmania* infection over such an extended period, or it could be a phenomenon that is more readily seen using the parasite strain we used in our study. Limits placed on sampling in human cases make it difficult to parse out how *Leishmania* infection induces hemophagocytosis. Hemophagocytosis sometimes accompanies pancytopenia, splenomegaly and anemia, three major symptoms of VL. Therefore, hemophagocytosis may have a role in the occurrence of each of these VL symptoms.

There are only isolated reports investigating anemia in rodent models of *Leishmania* infection. The only report on murine anemia deals with *L*. *tropica* infection [30], not infection by the *L*. *donovani* complex. *L*. *donovani*-induced anemia has only been reported in hamster models [31]. Although hamsters are regarded as a better model for VL pathology than mice, there are disadvantages to this model: a paucity of immunological reagents (e.g., antibodies) and animal handling and welfare (e.g., housing, generation of transgenic animals). In the present study, for the first time we demonstrated anemia in mice infected with *L*. *donovani* (Fig 1). The fact that anemia and hemophagocytosis, two clinical manifestations observed during human VL, can be achieved in a mouse model provides an avenue to investigate immunopathological mechanisms. In this study, we used *L*. *donovani* D10 strain, which is different from strains such as LV9 and LV82 that have been used by other researchers [31, 32]. If anemia/hemophagocytosis is D10-specific, a comparative study of those strains that induce anemia with those that do not may facilitate identification of parasite factors responsible for VL-associated anemia.

Animal models have been reported for *Salmonella-* [21], EB virus- [26] and *Trypanosoma brucei* [33]-associated hemophagocytosis. However, mechanisms underlying infection-associated hemophagocytosis appear to vary among these diseases. For example, infection of mice with *T*. *brucei*, a related trypanosomatid parasite and a causative agent of sleeping sickness, induces hemophagocytosis by macrophages [33]. However, unlike leishmaniasis, *T*. *brucei* remains extracellular in its mammalian hosts and infection-associated hemophagocytosis in trypanosomiasis is distinct from that observed with leishmaniasis since we demonstrated that direct infection by *Leishmania* amastigotes is an important factor leading macrophages to be hemophagocytic. This is supported by our finding that heavily infected macrophages were more phagocytic than those with no or low infection (Fig 3). In this respect, hemophagocytosis in VL seems to differ from that in EB virus or *T*. *brucei* infection where direct infection is a dispensable step in hemophagocytogenesis. On the other hand, Pilonieta *et al*. have reported that, in the case of *Salmonella* infection, the bacteria can be found in precisely the same types of hemophagocytes as those seen during *L*. *donovani* infection [21]. The authors speculated that *Salmonella* in hemophagocytes derive nutrient iron from commandeered erythrocytes. This speculation may be applied to *Leishmania* parasites because they do not have a heme synthetic pathway [34].

The finding that hemophagocytosis is most prominent in heavily infected macrophages provides an argument against the idea that damages/changes to erythrocytes contribute significantly to this phenomenon. Peripheral erythrocytes from the 24-week infected mice showed no apparent damage when examined by microscopy, and there was no apparent difference in osmotic fragility of erythrocytes from uninfected mice (S3 Fig). The other suggested major cause of hemophagocytosis is opsonization by autoantibodies [3]. The emergence of anti-erythrocyte antibodies has been reported in human VL patients [35, 36]. To test for this, we performed a direct agglutination test using anti-IgG to detect autoantibodies bound to the murine erythrocytes from *Leishmania-*infected mice. No agglutination was observed by the test (S4 Fig).

Identification of MGCs as the major hemophagocytes (Fig 4) also supports the idea that a macrophage abnormality, not an erythrocyte abnormality, is a principle factor leading to hemophagocytosis in infected mice and possibly human VL. There are several types of MGCs including Langhans giant cell and foreign body giant cell. Cytokines and T cells play key roles in MGC development: IFN-γ is a key factor for development Langhans giant cells [37], whereas IL-4 and IL-13 seem important for foreign body giant cell formation [38, 39]. Involvement of cytokines and lymphocytes in hemophagocytosis has been reported for various infectious diseases. IFN-γ and CD8^+^ T cells are central in hemophagocytosis during lymphocytic choriomeningitic virus infection [22]. Also, IFN-γ-deficient mice failed to manifest hemophagocytosis during *T*. *brucei* infection [33]. Both IFN-γ and IL-4 can cause anemia/hemophagocytosis through different pathways [40, 41]. *L*. *donovani-*infected macrophages may also receive extra signals including cytokines embarking them down the hemophagocytic pathway.

Positive signals of F4/80 confirmed the macrophage lineage of MGCs, whereas the MGCs had a reduced CD11b signal compared with non-infected CD11b^+^ macrophages (Fig 5). A recent study demonstrated that CD11b^low^ F4/80^+^ cells are involved in efferocytosis [42], which seems to be related to phagocytosis of unopsonized erythrocytes [43]. In fact, indications are that IFN-γ-induced hemophagocytosis follows a similar process as efferocytosis [40]. Also, MGCs were strongly stained with MOMA-2 antibody. Although the target molecule of MOMA-2 antibody has not been identified yet, the antibody seems to stain a small subpopulation of macrophages [44]. Lang *et al*. have previously reported that macrophages stained with MOMA-2 are the major host macrophages for *L*. *donovani* in the spleen [45], which is consistent our findings. Together, characterization of MCGs as F4/80^+^/CD11b^low^/MOMA-2^+^ may be useful for isolation of these cells by cell sorting, providing the means to further study their activation status and mechanisms involved in hemophagocytogenesis and hemophagocytosis.

## Supporting Information

S1 Fig Histology of the liver and bone marrow from *L*. *donovani*-infected mice. Representative images of the liver (A) and bone marrow (B) of *L*. *donovani*-infected mice at 24 weeks post-infection are shown. Arrows indicate macrophages harboring *L*. *donovani* amastigotes. Scale bar, 20 μm.
(TIF)

S2 Fig Increase of MOMA-2-positive macrophages in the spleen during *L*. *donovani* infection. Immunohistochemical staining of the spleen from naïve mice (A, C, E) or *L*. *donovani*-infected mice at 24 weeks post-infection (B, D, F) was performed. Tissue sections were stained with anti-F4/80 (A, B), MOMA-2 (C, D) and anti-CD11b (E, F) antibody, followed by counterstain with hematoxylin. Scale bars, 200 μm.
(TIF)

S3 Fig Osmotic fragility of erythrocytes from *L*. *donovani*-infected mice. Osmotic fragility of erythrocytes was examined according to a previous report (Stijlemans B *et al*., PLOS Negl Trop Dis, 2015, 9:e0003561) with modifications. Solutions containing 0.7, 0.6, 0.55, 0.5, 0.45, 0.4, 0.35, 0.3 and 0.2% (w/v) of NaCl (WAKO) were prepared and 200 μl of each solution were applied to V-bottom 96-well plates (Thermo Scientific). Two microliters of heparinized blood from either naïve or 24 week-infected mice were added to each well followed by gentle pipetting. After incubation at room temperature for 2 h, 100 μl of supernatant from each well after 1 ×*g* erythrocyte sedimentation was transferred to a new 96-well plate, and the absorbance at 550 nm was measured. Degree of hemolysis for each well was calculated according to the absorbance of wells for erythrocytes treated with deionized water as 100% hemolysis and that for PBS treatment as 0% hemolysis. Mean and SD of naïve mice (open circles, n = 5) or infected mice (closed circles, n = 5) are shown. Mean and SD of NaCl concentrations corresponding with 50% hemolysis were 0.45 ± 0.01% for naïve mice and 0.48 ± 0.02% for infected mice.
(TIF)

S4 Fig Undetectable IgG binding on erythrocytes from *L*. *donovani*-infected mice by direct agglutination test. The direct agglutination test was performed to examine the presence of IgG molecules bound to erythrocytes. Heparinized blood from either naïve or 24 week-infected mice was washed with DMEM twice by centrifugation at 200×*g* for 10 min, followed by resuspension with PBS to 25% hematocrit. Five microliters of the suspension was applied to each well of a V-bottom 96-well plate containing 100 μl of PBS and was mixed well. Then, 50 μl of goat anti-mouse IgG antibody (1/1,000 dilution in PBS, Fisher Scientific, Pittsburgh, USA) was added to each well. Erythrocyte agglutination was determined based on the presence of clumping, as assessed by visual examination. Individual wells shown in the lower panel correspond to individual mice in both groups (n = 5 for each group). As controls, blood from a naïve mouse was pretreated with indicated amounts of anti-mouse RBC monoclonal antibody (clone 34-3C, Hycult Biotech, Uden, Netherland) at room temperature for 1 h, before probing with anti-mouse IgG antibody.
(TIF)
