# Supplementary figures and images for: Hemophagocytosis in Experimental Visceral Leishmaniasis by Leishmania donovani
Source: PLoS Negl Trop Dis. 2016 Mar 4;10(3):e0004505. doi: 10.1371/journal.pntd.0004505 (PMC4778860; doi:10.1371/journal.pntd.0004505)

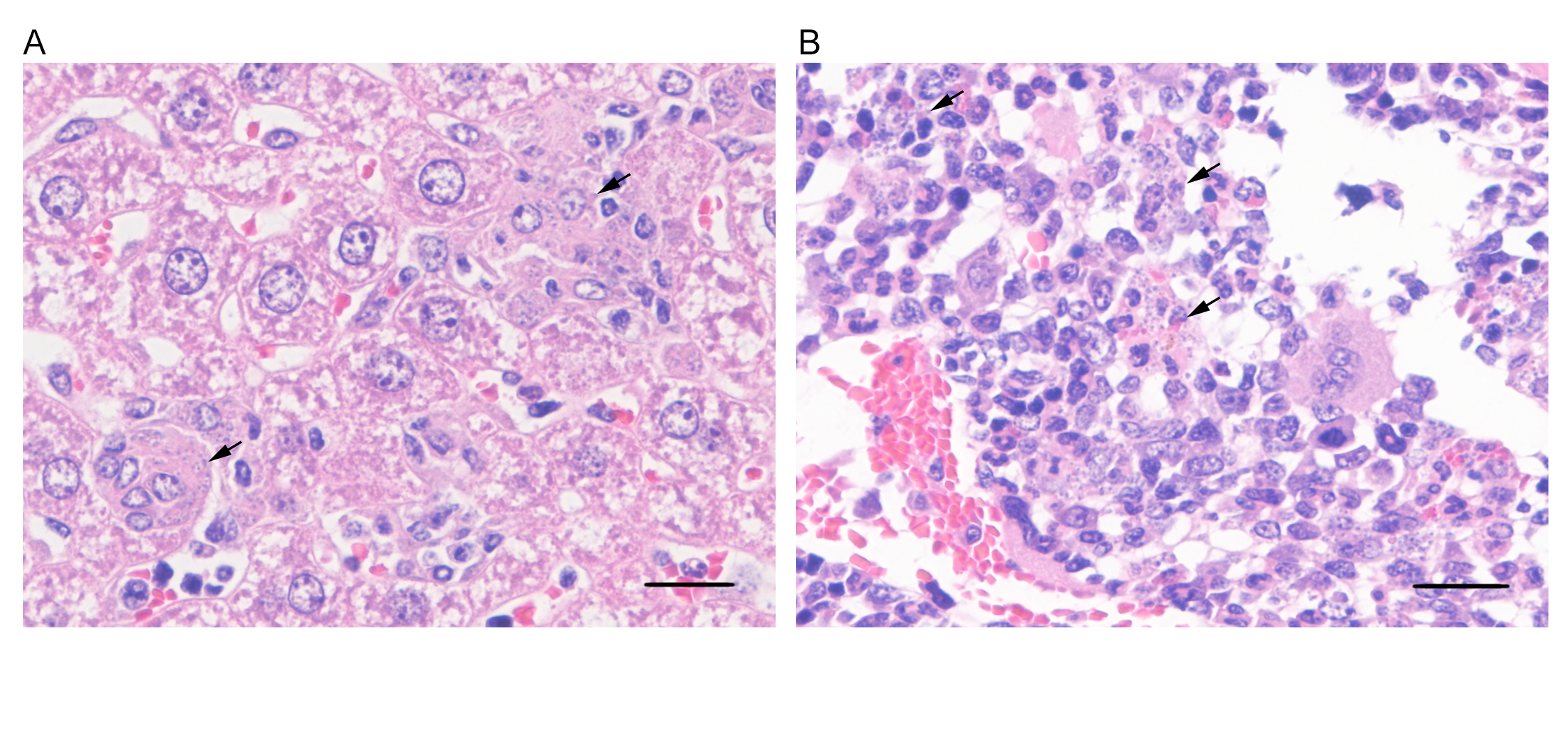

Supplement: S1 Fig — Representative images of the liver (A) and bone marrow (B) of L. donovani-infected mice at 24 weeks post-infection are shown. Arrows indicate macrophages harboring L. donovani amastigotes. Scale bar, 20 μm. (TIF) [file pntd.0004505.s001.tif]

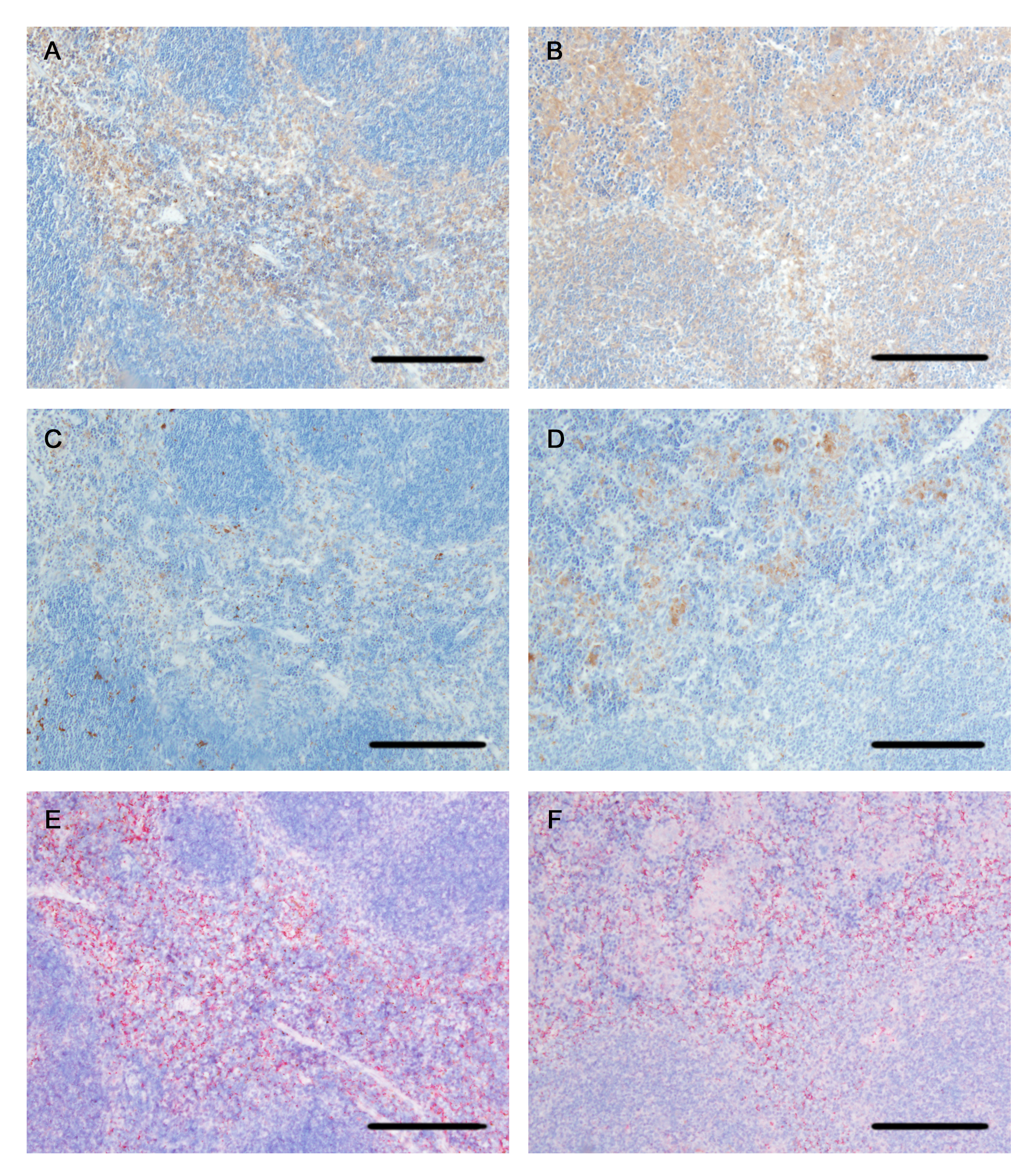

Supplement: S2 Fig — Immunohistochemical staining of the spleen from naïve mice (A, C, E) or L. donovani-infected mice at 24 weeks post-infection (B, D, F) was performed. Tissue sections were stained with anti-F4/80 (A, B), MOMA-2 (C, D) and anti-CD11b (E, F) antibody, followed by counterstain with hematoxylin. Scale bars, 200 μm. (TIF) [file pntd.0004505.s002.tif]

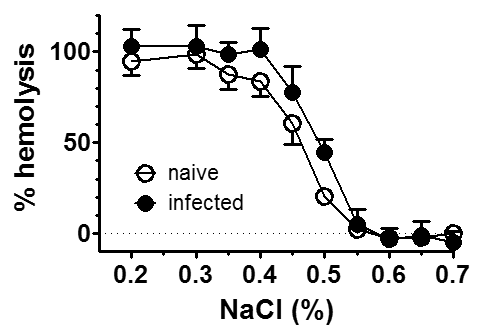

Supplement: S3 Fig — Osmotic fragility of erythrocytes was examined according to a previous report (Stijlemans B et al., PLOS Negl Trop Dis, 2015, 9:e0003561) with modifications. Solutions containing 0.7, 0.6, 0.55, 0.5, 0.45, 0.4, 0.35, 0.3 and 0.2% (w/v) of NaCl (WAKO) were prepared and 200 μl of each solution were applied to V-bottom 96-well plates (Thermo Scientific). Two microliters of heparinized blood from either naïve or 24 week-infected mice were added to each well followed by gentle pipetting. After incubation at room temperature for 2 h, 100 μl of supernatant from each well after 1 ×g erythrocyte sedimentation was transferred to a new 96-well plate, and the absorbance at 550 nm was measured. Degree of hemolysis for each well was calculated according to the absorbance of wells for erythrocytes treated with deionized water as 100% hemolysis and that for PBS treatment as 0% hemolysis. Mean and SD of naïve mice (open circles, n = 5) or infected mice (closed circles, n = 5) are shown. Mean and SD of NaCl concentrations corresponding with 50% hemolysis were 0.45 ± 0.01% for naïve mice and 0.48 ± 0.02% for infected mice. (TIF) [file pntd.0004505.s003.tif]

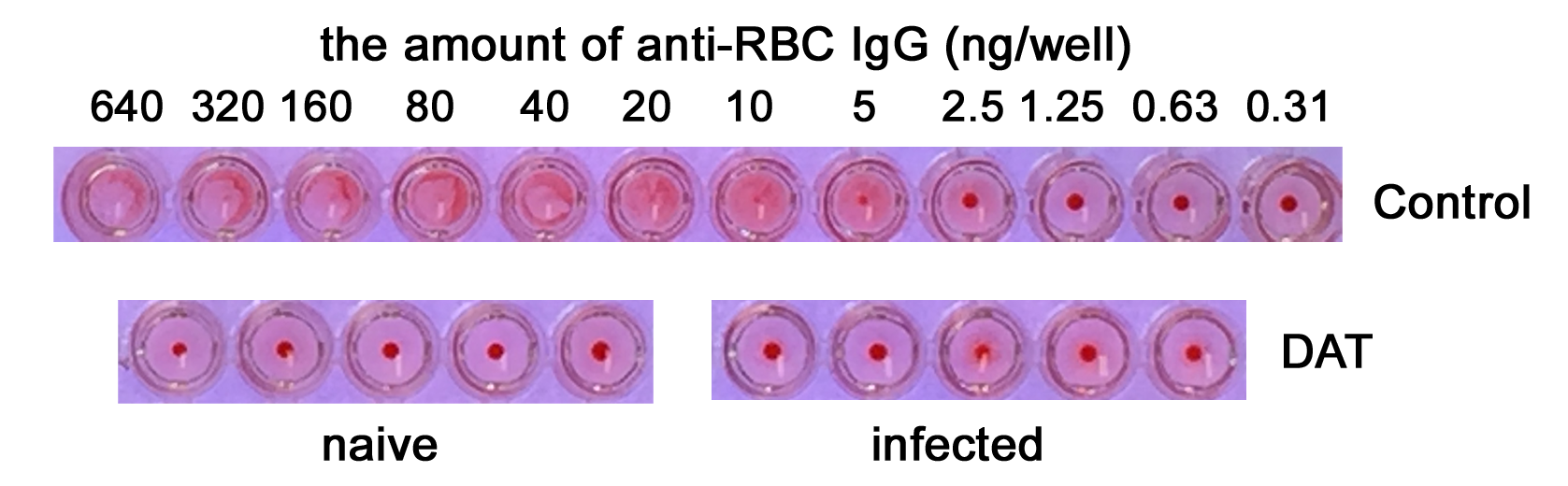

Supplement: S4 Fig — The direct agglutination test was performed to examine the presence of IgG molecules bound to erythrocytes. Heparinized blood from either naïve or 24 week-infected mice was washed with DMEM twice by centrifugation at 200×g for 10 min, followed by resuspension with PBS to 25% hematocrit. Five microliters of the suspension was applied to each well of a V-bottom 96-well plate containing 100 μl of PBS and was mixed well. Then, 50 μl of goat anti-mouse IgG antibody (1/1,000 dilution in PBS, Fisher Scientific, Pittsburgh, USA) was added to each well. Erythrocyte agglutination was determined based on the presence of clumping, as assessed by visual examination. Individual wells shown in the lower panel correspond to individual mice in both groups (n = 5 for each group). As controls, blood from a naïve mouse was pretreated with indicated amounts of anti-mouse RBC monoclonal antibody (clone 34-3C, Hycult Biotech, Uden, Netherland) at room temperature for 1 h, before probing with anti-mouse IgG antibody. (TIF) [file pntd.0004505.s004.tif]
